# Supplementary material for: Altered Gene Regulatory Networks Are Associated With the Transition From C3 to Crassulacean Acid Metabolism in Erycina (Oncidiinae: Orchidaceae)
Source: Front Plant Sci. 2019 Jan 28;9:2000. doi: 10.3389/fpls.2018.02000 (PMC6360190; doi:10.3389/fpls.2018.02000)
Supplement: Supplementary file 1 [file Data_Sheet_1.PDF]

Supplemental Figure 1

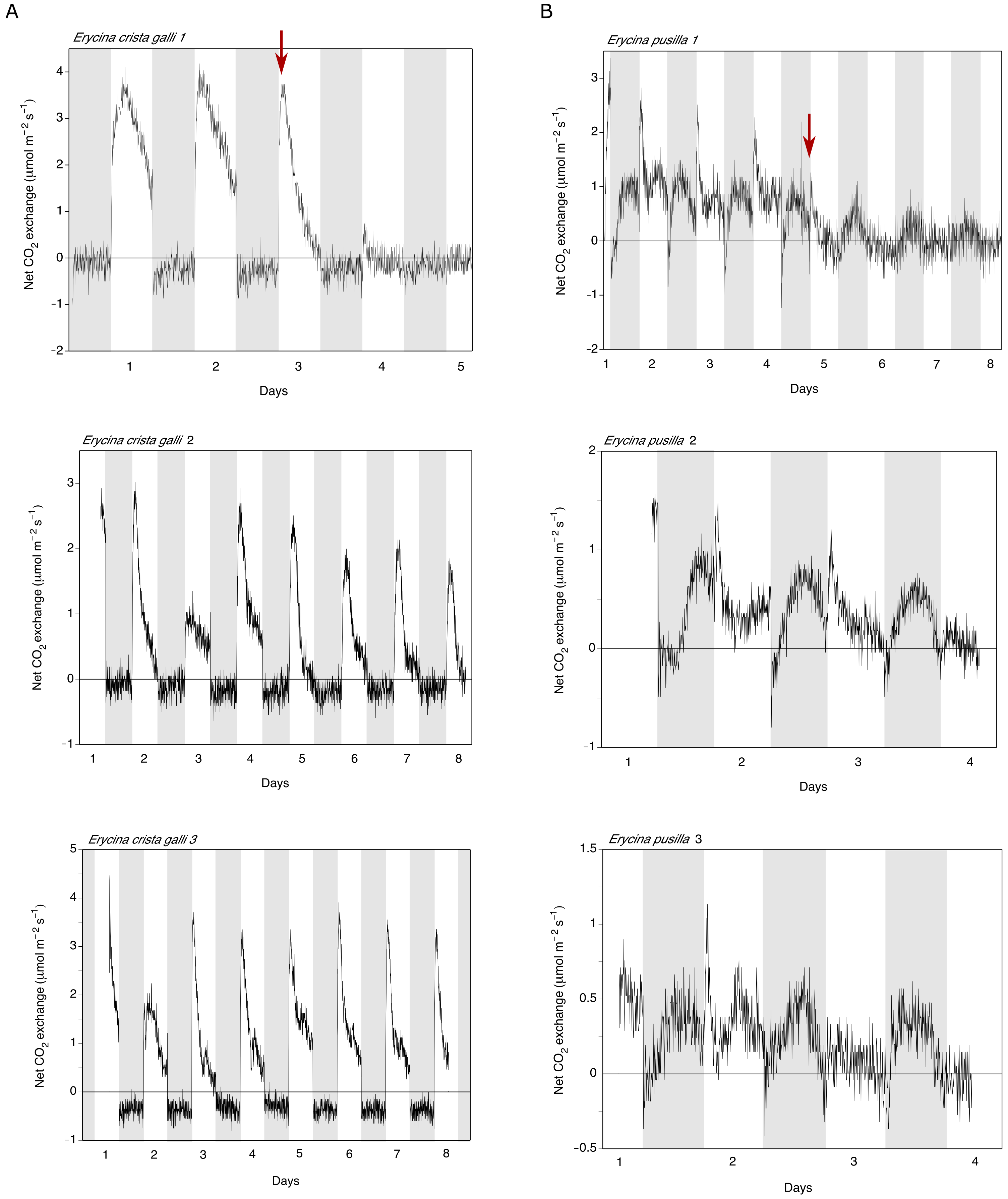

Supplemental Figure 1 - Gas exchange plots for E. crista-galli (A) and E. pusilla (B). The start of drought stress is indicated with red arrows for the two samples that were treated.
