# Supplementary material for: Altered Gene Regulatory Networks Are Associated With the Transition From C3 to Crassulacean Acid Metabolism in Erycina (Oncidiinae: Orchidaceae)
Source: Front Plant Sci. 2019 Jan 28;9:2000. doi: 10.3389/fpls.2018.02000 (PMC6360190; doi:10.3389/fpls.2018.02000)
Supplement: Supplementary file 2 [file Data_Sheet_2.PDF]

Supplemental Figure 2

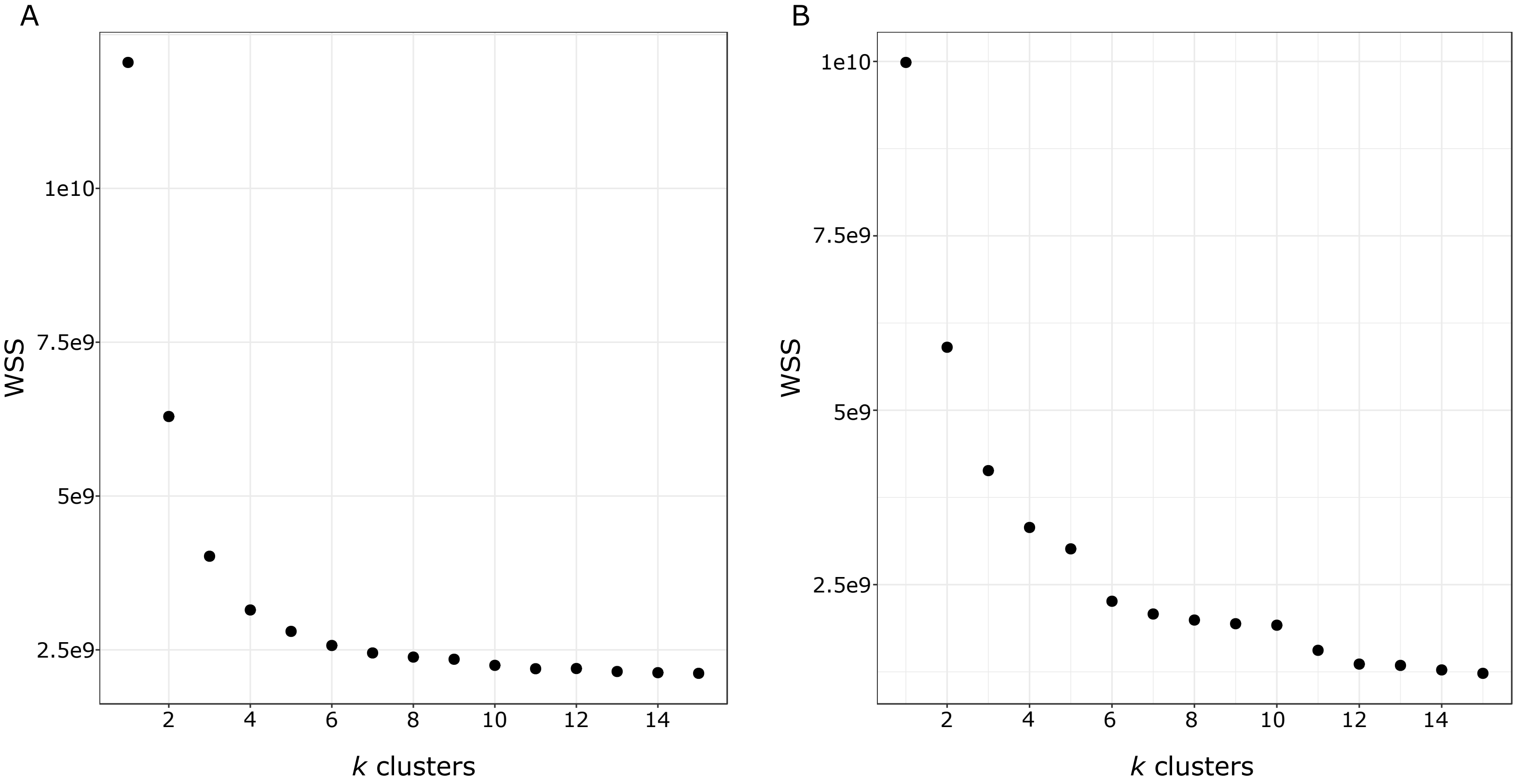

Supplemental Figure 2 - Within group sum of squares (WSS) calculations for each k betwen 1 and 15 for *E. pusilla* (A) and *E. crista-galli* (B).
