# Supplementary material for: Altered Gene Regulatory Networks Are Associated With the Transition From C3 to Crassulacean Acid Metabolism in Erycina (Oncidiinae: Orchidaceae)
Source: Front Plant Sci. 2019 Jan 28;9:2000. doi: 10.3389/fpls.2018.02000 (PMC6360190; doi:10.3389/fpls.2018.02000)
Supplement: Supplementary file 3 [file Data_Sheet_3.PDF]

Supplemental Figure 3

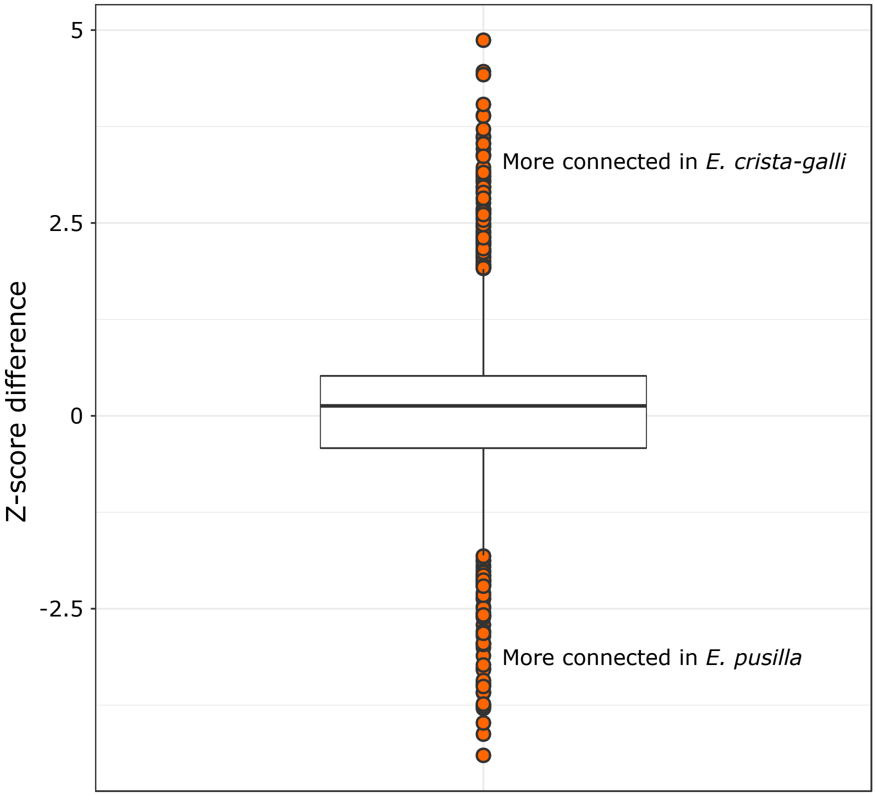

Supplemental Figure 3 - Number of directed edge outlier orthogroups. Z scores were first calculated within each species for each orthogroup (number of directed edges was the metric normalized, using the scale() function in R). Z scores for each orthogroup of *E. pusilla* were then subtracted from the Z score of the same orthogroup in *E. crista-galli*. Positive values therefore indicate increased connectivity in *E. crista-galli*, while negative values indicate i ncreased connectivity in *E. pusilla*. Orthogroups that were further investigated are those considered outliers by the boxplot() function in R, highlighted in orange.
